# Exploring the Mechanisms Linking Digital Leadership to Employee Creativity: A Moderated Mediation Model

**DOI:** 10.3390/bs15081024

**Published:** 2025-07-28

**Authors:** Mengxi Yang, Muhammad Talha, Shuainan Zhang, Yifei Zhang

**Affiliations:** 1 School of Economics and Management, University of Chinese Academy of Sciences, Beijing 100190, China; yangmengxi@ucas.ac.cn (M.Y.); m.talha89@mails.ucas.ac.cn (M.T.); zhangshuainan23@mails.ucas.ac.cn (S.Z.); 2 MOE Social Science Laboratory of Digital Economic Forecasts and Policy Simulation, University of Chinese Academy of Sciences, Beijing 100190, China

**Keywords:** digital leadership, employee creativity, knowledge sharing, innovation self-efficacy, technology readiness, social cognitive theory, social exchange theory

## Abstract

Employee creativity is essential for navigating digital disruption and maintaining organizational competitiveness; however, the mechanisms through which digital leadership fosters creativity remain underexplored. This study investigates the psychological and social processes through which digital leadership influences workplace creativity. Grounded in social cognitive and social exchange theories, the proposed model incorporates innovation self-efficacy and knowledge sharing as mediators and technology readiness as a moderator. Data were collected using a three-wave, time-lagged, multi-source survey design from 234 matched respondents, including employees and supervisors, across 20 business units in seven regional branches of a large Chinese organization undergoing digital transformation. The findings indicate that digital leadership significantly enhances employee creativity through the partial mediation of both innovation self-efficacy and knowledge sharing. Notably, the indirect effect through knowledge sharing was stronger, underscoring the critical role of collaborative processes in driving creativity. Furthermore, technology readiness positively moderates the effects of digital leadership on both mediators and amplifies the indirect effects on creativity. These findings provide valuable insights into how organizations can leverage digital leadership more effectively by aligning leadership strategies with employees’ psychological readiness and fostering a digitally supportive work environment.

## 1. Introduction

The rapid development and integration of emerging technologies, such as artificial intelligence (AI), big data, cloud computing, and blockchain, have made the digital economy a leading force behind high-quality economic development worldwide (S. Wang et al., 2024). As production methods, consumer expectations, and governance systems evolve, organizations unable to adapt to digitalization face the risk of obsolescence. In contrast, those guided by leaders who embrace innovation, foster risk-taking, and cultivate creativity will likely shape the future of competitive industries (Forbes, 2024). According to recent industry reports, workers increasingly trust AI, believing it enhances both productivity and work quality (Freshworks, 2024). Still, a significant portion of firms remain in the exploratory stages of digital adoption (National University, 2025), underscoring the untapped potential of digital tools to enable creativity and innovation (World Economic Forum, 2025).

Creativity, defined as the generation of novel and useful ideas (Torrance, 1972), is widely recognized as the cornerstone of sustainable organizational growth (Lee et al., 2019). It enables firms to navigate complexity, solve problems, and develop innovative solutions that foster long-term competitiveness. As the automation of routine tasks accelerates, employees are increasingly expected to perform cognitively complex, innovation-oriented roles (Haefner et al., 2021). Scholars have emphasized that employees’ creativity play a crucial role in executing and promoting corporate innovation activities compared to other organizational and individual factors (Volery & Tarabashkina, 2021). Thus, unlocking employees’ creative potential has become a critical concern for managers, especially in digitally transforming organizations.

Leadership remains a central factor in cultivating an environment conducive to creativity. Prior research has demonstrated the impact of various leadership styles—including transformational (Shafi et al., 2020; Al Harbi et al., 2019), ethical (Shafique et al., 2020), entrepreneurial (W. Cai et al., 2019), and inclusive leadership—on enhancing employee creativity. However, these traditional models may not sufficiently address the demands of leading in a digitally complex and fast-paced environment. In response, the concept of digital leadership has emerged, reflecting a leader’s ability to integrate digital technologies, inspire innovation, and facilitate digital transformation (Rakovic et al., 2024; Qiao et al., 2024). Digital leaders are increasingly recognized for their role in enhancing sustainable performance (Khaw et al., 2022), driving innovation (Y. Wang et al., 2025), fostering engagement (Li et al., 2024), digital intensity (Kludacz-Alessandri et al., 2025), and enabling creativity (Öngel et al., 2023). They combine strategic foresight, technological fluency, and a participative mindset to create environments in which knowledge sharing and value co-creation can thrive through open innovation and employee empowerment (Grimaldi et al., 2025).

Despite growing interest, the mechanisms through which digital leadership enhances employee creativity remain insufficiently understood (Öngel et al., 2023; Y. Wang et al., 2025). This study addresses this gap by investigating two key mediators, including innovation self-efficacy and knowledge sharing through the lens of social cognitive theory (Bandura, 2001) and social exchange theory (Blau, 1964). Social cognitive theory emphasizes the psychological mechanisms through which digital leadership influences creativity, particularly by enhancing innovation self-efficacy—an individual’s belief in their ability to generate creative outcomes (Bandura, 2001). Researchers have explained self-efficacy in the context of green innovation impacting economic performance (Alshebami, 2023) and work behavior (Uppathampracha & Liu, 2022). When employees perceive their leaders as empowering and digitally competent, they are more likely to develop confidence in their creative capabilities—resulting in higher motivation and problem-solving capacity (Gist & Mitchell, 1992; Luo et al., 2024).

In addition, this study incorporates knowledge sharing as a behavioral mechanism based on social exchange theory (Blau, 1964). Knowledge sharing, a voluntary behavior rooted in trust and reciprocity, is a fundamental enabler of collective creativity and innovation. It plays a vital role in expanding competitive advantage and enhancing organizational performance (Azeem et al., 2021; Olan et al., 2022). Employees are more inclined to exchange ideas when they perceive leadership to be supportive, fair, and participatory (Ahmad et al., 2023). Digital leaders enhance this process by leveraging collaborative technologies, promoting transparency, and breaking down silos between departments (Alajmi, 2022; Darvish et al., 2023). Though knowledge sharing has been studied in various organizational contexts such as the healthcare sector (Alshammary & Ali, 2024) to enhance creativity, its role as a mediating variable linking digital leadership to creativity remains underexplored.

Moreover, individual differences in technology readiness may influence how employees respond to digital leadership. While some employees quickly adapt to and master digital skills, others may feel anxious or resistant due to a lack of technical experience or unfamiliarity with new technologies. Defined as an individual’s propensity to embrace and effectively use new technologies (Parasuraman, 2000), technology readiness reflects psychological openness to digital change. SCT supports this by highlighting that employees with high levels of readiness are more likely to develop innovation self-efficacy, which boosts their ability to perceive digital leadership positively, engage with digital tools, and translate digital strategies into innovative outcomes (Cetindamar Kozanoglu & Abedin, 2021). Despite its relevance and that it is based on social cognitive theory, technology readiness has not been sufficiently examined as a moderator in leadership-creativity models—particularly in digitally transforming work environments.

In response to these literature gaps, this study develops a moderated mediation model to explore how digital leadership influences employee creativity via innovation self-efficacy and knowledge sharing and how these relationships are moderated by employees’ technology readiness. By integrating social cognitive and social exchange theories, the study advances a dual-process view of creativity in the digital workplace. It makes several contributions: first, by identifying key mediating mechanisms that explain how digital leadership drives creativity using social cognitive theory and social exchange theory, which have never been explored together in the literature (Zhao & Zhou, 2021; Y. Cai & Shi, 2022; Saleem et al., 2021; Meira & Hancer, 2021); second, by introducing technology readiness as a critical individual difference that shapes the effectiveness of digital leadership; and third, by using a multi-source, time-lagged design to enhance validity and causal inference. In addition, the study seeks to enrich the theoretical understanding of leadership in the digital age while offering practical guidance for organizations seeking to foster creativity through digital leadership and employee empowerment during periods of technological change.

## 2. Literature Review and Hypothesis Development

### 2.1. Digital Leadership and Employee Creativity

Digital leadership refers to the ability of leaders to create a clear and meaningful vision for the digital process and the ability to execute strategies to achieve it (Larjovuori et al., 2016). Such leaders utilize advanced information technology to influence the attitudes, emotions, thinking, behaviors, and performance of individuals and organizations (Avolio et al., 2000). Specifically, to meet the complex leadership needs of the digital age, digital leadership plays a crucial role in driving the digital transformation of enterprises (Brunner et al., 2023). Digital leaders encourage employees to actively innovate and adjust organizational structures to adapt to the digital environment (Kane et al., 2019). Moreover, digital leaders focus on redesigning business processes and creating an innovation culture through digital tools to promote the success of digital strategies (El Sawy et al., 2020) and organizational innovation (Ardi et al., 2020). Based on the resource-based view and dynamic capability theory, G. Wang et al. (2024) suggest that digital leadership relies on high-involvement human resource management practices and the improvement of employees’ dynamic capabilities to improve employees’ digital performance.

In the digital economy era, leaders have gradually realized that the traditional task allocation and supervision methods no longer adapt to the new work model (Antonopoulou et al., 2021). Leadership creates an environment conducive to innovation and development by promoting collaborative interaction and continuous learning among team members (Ahsan, 2025). Digital leadership provides employees with rich internal and external resources, efficient and transparent information acquisition channels, and open communication platforms through digital tools and online collaboration (Alajmi, 2022; Darvish et al., 2023). Employees gain easier access to cross-departmental and cross-domain information and extract inspiration from it, quickly iterate creative ideas, and form breakthrough solutions (El Sawy et al., 2020). In addition, digital leadership helps employees build expectations and confidence for the future by conveying a forward-looking digital vision. When employees see the huge potential behind digital transformation, they are more likely to actively propose innovative ideas (Guinan et al., 2019). Despite this, digital vision provides employees with specific innovation goals and encourages them to think about how to solve practical problems with digital technology, thereby boosting motivation for innovation and creativity (Öngel et al., 2023). Similarly, Zhu et al. (2022) indicates that digital leadership motivates employees to be more proactive in creative production and execution by optimizing work resources and reducing uncertainty. In addition, in the daily work context, the digital behavior and empowerment strategies of leaders during digital transformation can make it easier for employees to internalize innovative behaviors and achieve continuous improvement in creativity performance (Q. Lin & Yi, 2022). When leaders use digital means to promote organizational change and resource integration, employees are more likely to draw inspiration from multiple information sources (Fatima & Masood, 2024), influencing the self-efficacy and knowledge-sharing behavior. While prior research has emphasized the positive influence of digital leadership on employee creativity (Öngel et al., 2023; Zhu et al., 2022; Q. Lin & Yi, 2022; Fatima & Masood, 2024), the underlying psychological and behavioral mechanisms through which this effect occurs remain underexplored. Specifically, there is a lack of empirical studies examining the mediating roles of innovation self-efficacy and knowledge sharing as well as the moderating influence of technology readiness. Furthermore, research using an integrated moderated mediation framework to explain how and under what conditions digital leadership fosters employee creativity remains limited. Therefore, this study aims to address this gap by investigating both the mediating processes and the boundary conditions that shape the relationship between digital leadership and employee creativity.

### 2.2. Employees’ Innovation Self-Efficacy as a Mediator Between Digital Leadership and Employee Creativity

The theory of self-efficacy explains an individual’s subjective belief in their ability to complete a task or achieve a goal, which in turn affects their motivation, behavioral choices, and emotional responses (Bandura, 1977). In the context of innovation, innovation self-efficacy refers to an individual’s belief in their ability to generate innovative behavior in a specific situation (Tierney & Farmer, 2002). This belief encompasses four aspects of confidence, including the ability to generate new ideas, creativity in problem-solving, the capacity to help others realize new ideas, and the ability to find solutions to new problems. In addition, Gu and Peng (2010) further pointed out that innovative self-efficacy is not only reflected in an individual’s confidence in the results of their creative tasks but also in their beliefs about overcoming difficulties, solving problems, and achieving innovative goals. Social cognitive theory (Bandura, 1977) suggests that self-efficacy arises from four primary sources, including personal mastery experiences, vicarious experiences (such as observing others’ behaviors and outcomes), verbal persuasion (including affirmation and encouragement from others and society), and emotional arousal (such as psychological reactions when facing challenges).

Firstly, digital leadership provides employees with necessary information and resource support by establishing online knowledge bases, data analysis platforms, and internal social media (Kane et al., 2019). This enables employees to quickly locate solutions, reducing feelings of helplessness and uncertainty when pursuing innovation. External support and positive signals help employees form a positive cognition of their own innovation capabilities and improve their innovation self-efficacy. According to El Sawy et al. (2020), digital leadership actively uses emerging technologies, encourages experimentation, and tolerates failure in the organization, setting an example of innovation in the digital age for employees. Employees gain indirect experience by observing the flexibility and adaptability of leaders in digital environments. This external demonstration of success can strengthen employees’ self-efficacy. Moreover, digital leadership can monitor employees’ innovation activities using various digital tools and provide timely emotional counseling or targeted capacity training to address difficulties in the innovation process, thus reducing employees’ innovation anxiety to a certain extent.

Secondly, digital leadership, through transparent performance indicators, data-driven decision-making processes, and instant feedback mechanisms, enables employees to evaluate the effectiveness and value of their innovative behaviors more quickly and accurately (G. Wang et al., 2024). However, this continuous feedback builds a positive self-evaluation cycle for employees, enabling them to continuously improve their sense of innovation self-efficacy. In addition, by creating a flexible, open, and inclusive organizational culture (Alshammary & Ali, 2024), digital leadership enhances employees’ psychological security, reduces employees’ psychological barriers to innovation, and empowers employees to try new methods and tools. When employees are convinced that they have the ability to generate new and useful ideas, they will be more proactive in finding solutions to problems, iterating innovative ideas, and putting them into practice (Ng & Lucianetti, 2016). Thus, the proposed hypothesis is the following:

**H1:**  *Employees’ innovation self-efficacy mediates the impact of digital leadership on employees’ creativity.*

### 2.3. Knowledge Sharing as Mediator Between Digital Leadership and Employee Creativity

Knowledge sharing refers to the behavior of individuals or teams transferring information and experience related to tasks and technologies to solve problems faced by others or generate innovative ideas (S. Wang & Noe, 2010). Social exchange theory posits that knowledge sharing is a behavior rooted in reciprocity and mutual trust (Blau, 1964). Firstly, digital leadership, supported by digital technology and cultural guidance, fosters an environment where organizational members trust each other and are more inclined to share knowledge (Liang et al., 2016). By leveraging digital platforms, digital leadership transforms traditional knowledge-sharing methods, reducing both the difficulty and cost of such activities (Yang et al., 2025). These platforms enable seamless online communication, allowing employees to connect anytime, anywhere, making communication a fundamental part of daily organizational activities. Internal information systems facilitate cross-departmental and cross-level interactions, breaking down formal structural barriers to information flow (S. Wang & Noe, 2010). This leads to more frequent cross-professional and cross-regional communication, enabling employees to gain diverse perspectives and experiences, which fosters a broader knowledge-sharing network.

Secondly, digital leadership cultivates an open and transparent work environment, providing employees with better access to resources, technical support, and emotional encouragement (Kane et al., 2019). When employees perceive organizational or leadership support, they often feel a stronger sense of responsibility toward the team, reciprocating by sharing skills, experiences, and information with a more positive attitude (Bock et al., 2005). Moreover, digital leadership fosters a collaborative and reform-oriented atmosphere, encouraging cross-departmental cooperation and discussion (El Sawy et al., 2020). By modeling knowledge-sharing behaviors, digital leaders inspire employees to participate more actively. When articulating a vision of digitalization and sustainable development, digital leadership emphasizes self-organized learning and continuous innovation, aligning employees with shared goals and a sense of mission (Westerman et al., 2014). This leadership approach reinforces the belief that knowledge sharing is valued and rewarded within the organizational culture, motivating employees to engage in such activities (Cabrera & Cabrera, 2005).

Notably, knowledge sharing provides employees with tools and channels to access information from various professional domains. Through these interactions, tacit knowledge from different organizational fields is externalized and integrated, enabling individuals to identify innovation opportunities and solutions more rapidly (Nonaka & Takeuchi, 1996). Frequent knowledge exchange and integration allow employees to expand and reorganize existing knowledge, sparking creative thinking and practical innovation (Amabile, 2018). However, the literature highlights that the digital leadership promotes knowledge sharing by fostering an open technological culture and diverse communication channels, leading to high-quality knowledge-sharing network equipping employees with richer innovation resources and inspiration, enhancing their creativity (Perry-Smith & Shalley, 2003). Based on this, the following hypothesis is proposed:

**H2:**  *Knowledge sharing mediates the impact of digital leadership on employee creativity.*

### 2.4. Technology Readiness as Moderator in the Relationship Between Digital Leadership, Innovation Self-Efficacy, and Employee Creativity

The concept of technology readiness is defined as “the tendency of people to accept and use new technologies to achieve their personal and organizational goals” (Parasuraman, 2000). The Technology Readiness Acceptance Model highlights consumers’ optimism and innovativeness, significantly improving their perceived ease of use and usefulness of technology (C. H. Lin et al., 2007). For example, consumers with high technology readiness showed a higher willingness to use unmanned convenience stores, while consumers with low technology readiness faced more challenges (H. J. Park & Zhang, 2022). In virtual tourism scenarios, technology readiness not only directly affects consumers’ acceptance of augmented reality (AR) technology but also further strengthens destination loyalty through the humanized experience of AR (Kim et al., 2020). Significantly, optimism and innovation in technology readiness enhance teacher engagement compared to discomfort and insecurity (Joseph et al., 2021). Moreover, Khoza et al. (2024) suggest that technology readiness positively impacts work engagement, with employees’ optimism and innovation levels serving as key drivers that need to be considered when introducing technology in the workplace.

Technology readiness influences employee’s inclination toward implementing new technology solutions and encourages participation in tasks that involve self-directed learning while also affecting the psychological empowerment resulting from digital leadership (Parasuraman & Colby, 2015). Employees with high technical readiness can use the resources provided by digital leadership more effectively (Khoza et al., 2024). Such employees have a shorter learning curve for new tools, feel less uncertainty about the technical environment, and are more likely to form a positive perception of the digital environment. While these individuals also depict more adaptive performance and higher levels of psychological well-being due to the meaningfulness of their work (Abdul Hamid, 2022). Conversely, employees with low technology readiness are often surrounded by fear or a lack of trust in technology (Parasuraman, 2000; Joseph et al., 2021). Even when digital leaders provide platforms and learning opportunities, these employees struggle to develop positive perceptions due to resistance, thereby diminishing the positive impact of digital leadership. Likewise, when interacting with digital robots, the low-technology-readiness employees influence psychological well-being (Sehgal et al., 2025). Despite these challenges, digital leadership promotes creativity among staff by underpinning innovation self-efficacy (Amabile & Pratt, 2016), which serves as a key mediator between leadership and creative performance in this study. Based on social cognitive theory, employees with a high level of technology readiness are more open to digital devices, and with effective digital leadership, they can translate their innovative self-efficacy (psychology) into creative performance (H. J. Park & Zhang, 2022). In contrast, lower-technology-readiness employees face challenges in utilizing innovation-supportive resources from digital leadership, which weakens the mediating effect of innovative self-efficacy (World Economic Forum, 2025). Therefore, the following hypotheses are proposed:

**H3a:**  *Technology readiness positively moderates the impact of digital leadership on employee innovation self-efficacy.*

**H3b:**  *Technology readiness positively moderates the mediating effect of employee innovation self-efficacy.*

### 2.5. Technology Readiness as Moderator in the Relationship Between Digital Leadership, Knowledge Sharing and Employee Creativity

The level of technology readiness is an important indicator of how well employees can work with digital tools and foster knowledge sharing within an organization, as explained by the Technology Acceptance Model (TAM) (Davis, 1989; Kaewsaeng-On et al., 2022). The TAM suggests that perceived usefulness and ease of use drive technology acceptance, with employees who have high readiness viewing digital tools—such as knowledge management systems and collaboration platforms—as valuable and accessible, thereby facilitating creative knowledge sharing (C. H. Lin et al., 2007). This process enhances organizational innovation through effective knowledge circulation (Davenport & Prusak, 1998). On the other hand, the employees with low technology readiness tend to resist digital technologies, perceiving them as complex or untrustworthy, which hinders knowledge sharing (Parasuraman, 2000; Khoza et al., 2024). According to Cimbaljević et al. (2024), technology readiness influences employee intention and attitude towards technology. In the context of AI technology, technology readiness positively influences employee behavior to adopt AI, enhancing the organizational performance (Felemban et al., 2024). In our study, based on social cognitive theory, employee technology readiness moderates digital leadership’s role in promoting knowledge sharing (behavior). Higher readiness strengthens leadership’s impact by fostering positive perceptions of usefulness and ease of use of digital tools. At the same time, employees with relatively high technology readiness can quickly understand and use digital tools to share knowledge. This, in turn, provides employees with more innovative inspiration and leads to the promotion creativity in the workplace (Al-Husseini, 2024). However, employees with low technology readiness make inappropriate use of knowledge-sharing platforms, which limits the flow of necessary information and reduces support for employee creativeness (Alshammary & Ali, 2024; Fischer & Döring, 2022). Thus, the following hypotheses are proposed:

**H4a:**  *Technology readiness positively moderates the impact of digital leadership on knowledge sharing.*

**H4b:**  *Technology readiness positively moderates the mediating role of knowledge sharing.*

### 2.6. Theoretical Framework

**Figure 1:**
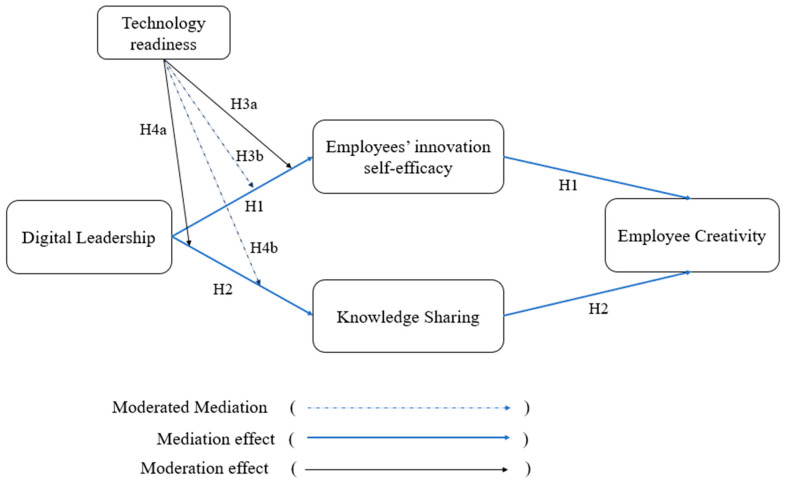
Research model.

The conceptual model of this study is based on Social Cognitive Theory (SCT) (Bandura, 2001) and Social Exchange Theory (SET) (Blau, 1964), offering a dual-theoretical lens to explain how digital leadership enhances employee creativity (see Figure 1). SCT emphasizes that individuals develop behaviors through observational learning and cognitive processing of environmental cues, with self-efficacy serving as a central mechanism driving engagement and adaptability (Wood & Bandura, 1989). In digitally transforming organizations, digital leaders play a crucial role in shaping these cognitive processes by modeling innovative behaviors, offering encouragement, and empowering employees with technological tools. This leadership style enhances innovation self-efficacy, which in turn promotes creative performance (Salanova et al., 2005). While SET suggests that leadership support fosters reciprocal behaviors such as knowledge sharing, which strengthens social capital and collaborative innovation. Employees who perceive digital leadership as supportive are more inclined to share knowledge, generating collective insights that fuel creativity. Together, SCT and SET support the partial mediating roles of innovation self-efficacy (psychological) and knowledge sharing (behavioral) in linking digital leadership to employee creativity. Additionally, SCT’s emphasis on individual differences (Bandura, 2001) allows our study to examine how technology readiness moderates these psychological and behavioral mechanisms, amplifying the effects of digital leadership. It proposes that employees with higher readiness are more responsive to digital leadership efforts—amplifying its effects on psychological and behavioral mechanisms that ultimately drive creativity.

The following model illustrates the hypothesized relationships between the investigated variables.

## 3. Research Methodology

### 3.1. Research Design

The current study employs a quantitative, time-lagged research design grounded in the positivist research paradigm, as described by Y. S. Park et al. (2020), which emphasizes objectivity, quantitative measure, and generalizability. A deductive approach was adopted, drawing upon social cognitive theory and social exchange theory to formulate hypotheses concerning the mediating and moderating mechanisms between digital leadership and employee creativity. Given the complexity of the proposed model, the quantitative approach was deemed appropriate (Creswell & Creswell, 2017), as it enabled the structured testing of hypotheses through statistical procedures. In addition, the time-lagged design enhanced the robustness of the study by reducing common method bias and allowing for greater inference of causal relationships among the variables (Podsakoff et al., 2012).

### 3.2. Sampling and Data Collection

We collected data from employees and their supervisors from Company Z using a three-wave, time-lagged survey design conducted over a period of four months. Company Z is a large organization in China undergoing digital transformation. The non-probability purposive sampling technique helped to recruit respondents from 20 business units across seven regional branches, ensuring diversity in departmental roles and levels of exposure to digital initiatives. The purposive sampling enabled the selection of respondents with direct experience relevant to the study’s constructs (Campbell et al., 2020), thereby enhancing the contextual accuracy and theoretical alignment of the data. In addition, the study’s multi-source survey design allowed employees to complete surveys in the first two waves while supervisors evaluated employee creativity in the final wave, hence reducing respondent consistency bias and enhancing internal validity (Podsakoff et al., 2003).

The surveys were administered electronically via Questionnaire Star and Qualtrics. To accurately match responses across survey waves while maintaining respondents’ anonymity, respondents were instructed to use the last five digits of their mobile phone numbers as login identifiers. Based on the suggestion of Van Teijlingen and Hundley (2002), a pilot study including 20 participants was conducted before the formal data collection to test the clarity, reliability, and contextual fit of the questionnaire. Grounded on the feedback, minor revisions were made to improve item wording and structure. However, in the first wave (December 2024), data on digital leadership, technology readiness, and demographic variables were collected, resulting in 279 valid responses from 319 distributed surveys (87.5% response rate). In the second wave (January–February 2025), measures of innovative self-efficacy and knowledge sharing were administered, yielding 266 valid responses (95.3%). In the third wave (March 2025), employee creativity was assessed through supervisor ratings, resulting in total of 234 matched responses (88.0%). Moreover, the male and female participants were 73.9% and 26.1%, respectively. The average age of respondents was approximately 30.6 years. On average, participants had worked at the company for 6.39 years, with an average overall length of service of 7.70 years. In terms of education, approximately 80% of respondents had an undergraduate degree.

### 3.3. Variable Measurement

All items were rated on 5-point Likert scale (1 = Strongly disagree, 5 = Strongly agree) unless otherwise noted (Appendix A).

Digital Leadership. Participants accessed the digital leadership using six items adapted from Zeike et al. (2019). Sample item includes “My leader is very interested in using digital technologies and tools” (α = 0.882).

Innovation self-efficacy. We accessed the innovation self-efficacy using the three-item scale adapted from Tierney and Farmer (2002). Sample item includes “I am confident in my ability to use creativity to solve problems” (α = 0.779).

Knowledge Sharing: Participants accessed this construct using six item scale adapted from Lu et al. (2006). Sample item includes “I share useful work experience and insights with everyone” (α = 0.877).

Employee Creativity: The study operationalized employee creativity using four item scale adapted from Baer and Oldham (2006). Sample item includes “I often come up with creative solutions to problems at work” (α = 0.808).

Technology Readiness: Participants accessed this construct using the 16-item scale adapted from Parasuraman and Colby (2015). Sample item includes “Technology gives people more control over their daily lives” (α = 0.960).

Control variable. The current study followed the empirical approach of Farmer et al. (2003) by including age, gender, education level, years of work in the company, and length of service as control variables. These demographic factors were controlled to account for their potential impact on employee creativity and to ensure that the effects of the main study variables were not confounded. The previous literature suggests that age and gender were found to correlate with individual creativity (Chua & Jin, 2020; Binnewies et al., 2008). Research indicates that the level of education indicates the level of knowledge of the particular domain and analytical ability, both being the highly essential inputs to creative performance (Tierney & Farmer, 2002). Previous research also emphasizes that the number of years spent in an organization and the total experience can also be factors that determine employee level of creativity through how well one is acquainted with organizational norms and problem-solving trends and being innovative (Oldham & Cummings, 1996). Therefore, we controlled for age (in years), gender (Male = 1, Female = 2), education level (Below high school = 1, High school = 2, College = 3, Bachelor’s degree = 4, Master’s degree = 5, Doctorate = 6), work tenure in the company (in years), and length of service (in years) in our study analysis.

In addition, suggested by Field (2024), we further evaluated the quality of each item in the scale by calculating the Corrected Item–Total Correlation (CITC) value of reliability analysis of each item (the correlation coefficient between the corrected item and the total score). The minimum CITC coefficient of each item in the scale was 0.653, which is greater than 0.5, indicating that there was a good correlation between each item and the overall score of the scale, and the reliability of the scale met the research requirements.

## 4. Analyses and Results

The mean, standard deviations, and correlations results are shown in Table 1. To evaluate the distinctiveness of the study’s constructs, a series of confirmatory factor analyses (CFAs) were conducted (Table 2). The hypothesized five-factor model, which treated digital leadership, knowledge sharing, innovation self-efficacy, employee creativity, and technology readiness as separate latent constructs, demonstrated the best fit to the data (χ^2^ = 762.160, df = 550, χ^2^/df = 1.386, RMSEA = 0.041, CFI = 0.956, TLI = 0.952). In contrast, the one-factor model, in which all variables were combined into a single latent factor, exhibited the poorest fit (χ^2^ = 2444.430, df = 560, χ^2^/df = 4.365, RMSEA = 0.120, CFI = 0.610, TLI = 0.585). However, the progressive decline in model fit across the alternative models provides strong support for the discriminant validity of the five key constructs, which was not achievable through other methods like EFA. In addition to the study’s multi-source data and time-lagged design, the result of Harman’s single-factor test indicates that a single factor accounted for only 33.11% of the variance, which is below 50%, confirming that the data are free of common variance bias (Podsakoff et al., 2003).

### Hypothesis Testing

Following Hayes’ (2018) work, this study estimated the moderated mediation model 7 of PROCESS macro. The current study selected the PROCESS macro, as it efficiently handles complex models with multiple mediators and moderators, enabling the simultaneous testing of the effects of innovation self-efficacy, knowledge sharing, and technology readiness. Unlike Structural Equation Modeling (SEM), which requires more complex assumptions and separate models, PROCESS provides a flexible and clear approach for analyzing conditional relationships, ensuring straightforward and interpretable results aligned with our theoretical framework.

The regression results indicate significant relationships among constructs, controlling for gender, age, education, work experience, and tenure (Table 3). The results show that digital leadership significantly predicted innovation self-efficacy (β = 0.266, SE = 0.076, *p* < 0.01), knowledge sharing (β = 0.328, SE = 0.069, *p* < 0.01), and employee creativity (β = 0.296, SE = 0.072, *p* < 0.01). In addition, both innovation self-efficacy (β = 0.176, SE = 0.062, *p* < 0.01) and knowledge sharing (β = 0.224, SE = 0.069, *p* < 0.01) had significant positive effects on employee creativity. Technology readiness was also positively associated with innovation self-efficacy (β = 0.224, SE = 0.072, *p* < 0.01) and knowledge sharing (β = 0.221, SE = 0.066, *p* < 0.01).

**Figure 2:**
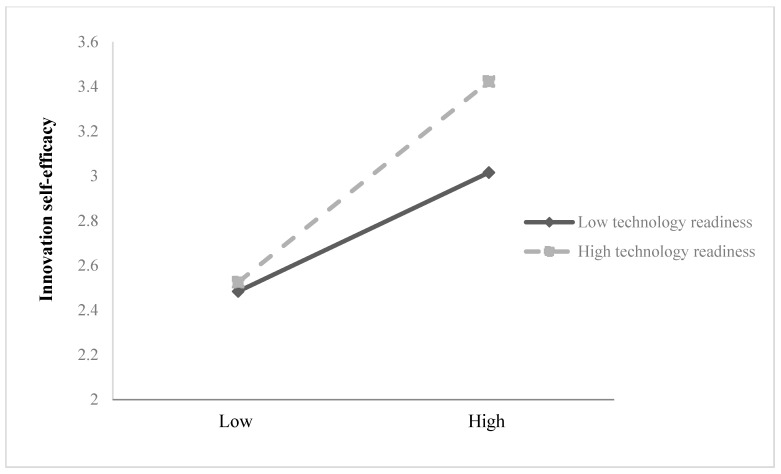
Interaction between digital leadership and technology readiness in predicting innovation self-efficacy.

**Figure 3:**
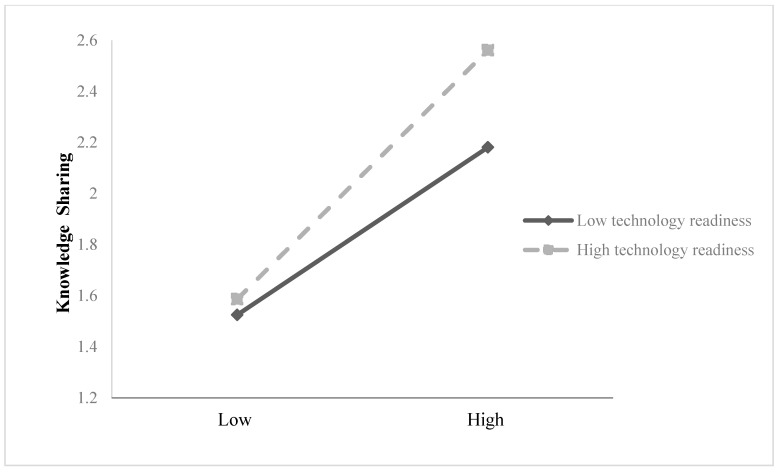
Interaction between digital leadership and technology readiness in predicting knowledge sharing.

Significantly, for the mediation analysis, the indirect effect of digital leadership on employee creativity through innovation self-efficacy was statistically significant (β = 0.079, SE = 0.035, LLCI = 0.017, ULCI = 0.154), indicating that innovation self-efficacy partially mediates this relationship (Table 4). Therefore, Hypothesis 1 is supported. Similarly, the indirect effect of digital leadership on employee creativity through knowledge sharing was also significant (β = 0.110, SE = 0.041, LLCI = 0.033, ULCI = 0.194), providing support for Hypothesis 2 (Table 4). In contrast, regarding moderation in Table 3, the interaction between digital leadership and technology readiness (DL × TR) was found to be significant in predicting innovation self-efficacy (β = 0.183, SE = 0.073, *p* < 0.05). Figure 2 presents the interaction effect, suggesting that the positive effect of digital leadership on innovation self-efficacy is stronger when employees exhibit higher levels of technology readiness. Thus, Hypothesis 3a is supported. Additionally, Table 3 also indicates that the interaction between digital leadership and technology readiness (DL × TR) significantly predicts knowledge sharing (β = 0.159, SE = 0.066, *p* < 0.05). Figure 3 presents the interaction effect, which predicts that the technology readiness strengthens the positive effect of digital leadership on knowledge sharing, hence leading to acceptance of Hypothesis 4a.

Interestingly, the moderated mediation analysis further confirmed the conditional nature of the indirect effects. The index of moderated mediation was significant for both pathways (Table 4). Specifically, we found that the conditional indirect effect of digital leadership on employee creativity via innovation self-efficacy was stronger at higher levels of technology readiness (β = 0.032, SE = 0.019, LLCI = 0.002, ULCI = 0.074), supporting Hypothesis 3b. Similarly, the conditional indirect effect through knowledge sharing was also significant (β = 0.036, SE = 0.020, LLCI = 0.002, ULCI = 0.080), providing support for Hypothesis 4b.

## 5. Discussion

The findings offer robust empirical support for the proposed moderated mediation model, shedding light on how and under what conditions digital leadership can enhance creativity within digitally transforming organizations. The findings reveled that the digital leadership had a significant impact on employee creativity. More importantly, this relationship was partially mediated by innovation self-efficacy and knowledge sharing. While the indirect effect through knowledge sharing was slightly stronger than through innovation self-efficacy, highlighting the crucial role of social and collaborative mechanisms in the creativity process. In digital context, creativity is not only a result of individual innovation but also of collective creativity, where knowledge sharing enhances collaboration, facilitates the exchange of ideas, and enables teams to develop more innovative solutions (Karakaya & Demirkan, 2015). Compared to knowledge sharing, innovation self-efficacy, though important in fostering individual creativity, was less impactful in driving innovation at the organizational level, where teamwork and collaboration are crucial.

In addition, the results demonstrate that technology readiness significantly moderates the effects of digital leadership on innovation self-efficacy and knowledge sharing, with the relationships being more pronounced for employees with higher levels of technology readiness. The moderated mediation analysis further revealed that technology readiness also moderates the mediating pathways, reinforcing its role as a critical boundary condition in digitally enabled creative work. These findings have significant implications for management research and practice.

### 5.1. Theoretical Implications

This study offers several important contributions to the literature on digital leadership, employee creativity, and organizational behavior. First, it advances current understanding by developing and empirically testing a moderated mediation model that explains how digital leadership enhances employee creativity through two distinct mechanisms, including innovation self-efficacy and knowledge sharing. While previous studies have established digital leadership as a driver of innovation outcomes (e.g., Öngel et al., 2023; Y. Wang et al., 2025), the underlying processes through which this influence operates remain underexplored. By identifying innovation self-efficacy and knowledge sharing as partial mediators, the study integrates social cognitive theory (Bandura, 2001) and social exchange theory (Blau, 1964) to explain both the psychological and behavioral pathways through which digital leadership shapes creative outcomes. This contributes to a richer, more process-oriented understanding of leadership effectiveness in digitally transforming workplaces.

Specifically, in our study context, SCT explains that innovation self-efficacy functions as a psychological driver by shaping employees’ belief in their ability to innovate. Digital leadership, through feedback, support, and the modeling of creative behaviors, enhances this self-belief, motivating employees to engage in innovative behaviors, which ultimately foster creativity. SET, on the other hand, emphasizes that knowledge sharing is a critical social mechanism that facilitates creativity. Leadership behaviors such as trust, reciprocity, and open communication create a supportive environment that encourages employees to share knowledge, thus enhancing collaboration and amplifying collective creativity.

These findings have significant implications for leadership processes in digital contexts. Digital leadership plays a critical role in fostering knowledge sharing, which in turn enhances creativity and innovation across teams. The results suggest that leaders in digital environments should focus on promoting collaborative behaviors and creating structures that encourage the exchange of ideas rather than solely focusing on building individual self-efficacy.

Second, this study introduces technology readiness as a critical moderating variable, offering insights into the conditions under which digital leadership becomes more or less effective. While technology readiness has been widely studied in relation to technology adoption and innovation acceptance (Parasuraman, 2000), its role in moderating leadership-driven outcomes has received limited attention. The results demonstrate that technology readiness not only strengthens the direct effects of digital leadership on innovation self-efficacy and knowledge sharing but also amplifies the indirect effects on employee creativity. This finding also responds to the calls for more context-sensitive models by showing that employees’ technological orientation plays a key role in shaping how leadership influences workplace behavior and performance (Cetindamar Kozanoglu & Abedin, 2021).

Third, this study contributes to the creativity literature (Öngel et al., 2023; Zhu et al., 2022; Q. Lin & Yi, 2022; Fatima & Masood, 2024) by shifting attention from traditional leadership paradigms, such as transformational or inclusive leadership, toward the more context-specific and technologically attuned construct of digital leadership. The findings underscore the importance of digital leaders’ ability to inspire, empower, and facilitate innovation in environments characterized by technological change. Furthermore, the evidence that both individual beliefs (innovation self-efficacy) and interpersonal dynamics (knowledge sharing) partially mediate the effect of digital leadership on creativity reinforces the view that creative behavior is shaped by internal cognition and social exchange.

Not only that, the study offers methodological contributions by employing a time-lagged, multi-source research design, thus mitigating common method bias and strengthening causal inference. By collecting data across three phases from both employees and their direct supervisors, this study enhances the internal validity of the proposed model and sets a precedent for future studies examining complex moderated mediation frameworks in organizational contexts.

### 5.2. Practical Implications

An important question for organizations undergoing digital transformation is how to effectively enhance employee creativity through leadership. The results of this study demonstrate that digital leadership fosters creativity through two distinct mechanisms: innovation self-efficacy and knowledge sharing, both functioning as partial mediators. Notably, the indirect effect through knowledge sharing was stronger (β = 0.1096), emphasizing the central role of collaborative behavior and social exchange in the creativity process. The study findings yield several actionable insights. First, organizations should adopt leadership practices that build employees’ confidence in their creative potential. Managers can strengthen innovation self-efficacy by implementing mentorship programs and feedback sessions and providing autonomy in decision-making. Recognizing creative contributions and fostering a climate that supports experimentation and tolerates failure (Huang et al., 2024) are also essential for boosting self-efficacy and fostering an innovation-friendly environment.

Second, the results highlight that cultivating a culture of open knowledge sharing is essential given its comparatively stronger mediating effect. To achieve this, organizations should implement digital collaboration platforms such as Slack, Microsoft Teams, or other tools that enable real-time information exchange. Reward systems for idea exchange, along with initiatives like cross-departmental projects or lunch-and-learn sessions, can break down barriers to communication and encourage a culture of knowledge sharing (Ungureanu et al., 2021). The findings also imply that leaders in digital environments should focus on promoting collaborative behaviors and creating structures that encourage the exchange of ideas rather than solely focusing on building individual self-efficacy.

In addition, the study reveals that technology readiness significantly moderates the effects of digital leadership on both mediators, with a stronger interaction effect observed in the innovation self-efficacy pathway (β = 0.1829). Managers should recognize that employees with higher technology readiness respond more positively to digital leadership—demonstrating greater creative confidence and a stronger inclination to share knowledge. To leverage this, managers could use targeted training sessions and digital tools to empower employees to become more comfortable with new technologies (Handrich & Otterbach, 2024). These actions will ensure that employees are better equipped to adapt and innovate in a digital-first environment.

In addition, the moderated mediation analysis shows that the indirect effects of digital leadership on creativity are amplified for high-readiness employees, particularly through knowledge sharing (β = 0.0356). At the same time, managers should not overlook the continued importance of innovation self-efficacy. Actively strengthening employees’ creative confidence through departmental collaborations focused on skill development, creative problem-solving challenges, or peer learning groups remains a vital lever for maximizing the impact of digital leadership in innovation-driven environments.

Interestingly, the findings also make it clear that digital leadership alone is not sufficient. Organizations must evaluate and support employees’ technology readiness to ensure they are fully equipped to engage with digital strategies. During digital transformation, tailored interventions—such as digital upskilling programs, mentoring, or phased onboarding aligned with individual comfort levels—can help bridge readiness gaps (Vial, 2021). These initiatives should be incorporated into employee development plans, ensuring that the right level of support is provided for each employee. Ultimately, digital leadership is most effective when combined with readiness-based enablement, ensuring that all employees are empowered to contribute creatively within dynamic, technology-driven environments.

### 5.3. Limitations and Future Research Directions

This study also has a few limitations. First, while this work was guided by social cognitive theory and social exchange theory, future studies could explore the proposed relationships through the lens of alternative theoretical frameworks—such as the Technology Acceptance Model (TAM), Self-Determination Theory, or Resource-Based View—to provide different perspectives on how digital leadership influences creativity. Such theoretical diversification could enrich our understanding of the motivational, technological, and organizational mechanisms involved in shaping employee creativity. Additionally, incorporating multiple theoretical lenses can reduce the limitations imposed by any one framework and allow for a deeper understanding of the complex dynamics of digital leadership.

Second, while technology readiness served as a meaningful moderator in this study, other individual or contextual factors may also condition the effectiveness of digital leadership. Potential moderators such as digital literacy, psychological safety, or perceived organizational support may provide further insight into how and when digital leadership is most effective. These factors are particularly relevant given the diversity in employee technological skills and perceptions of workplace safety, which can significantly affect how leadership influences creativity. Future research could incorporate these variables to expand the boundary conditions of the model, offering a richer understanding of the conditions under which digital leadership is most impactful.

Third, the generalizability of the findings is limited due to the specific sample and context. The study focused on a company undergoing digital transformation, which may not be applicable to all types of companies, industries, or cultural settings. Future research should include cross-industry comparisons, diverse cultural contexts, and experimental designs to explore how varying organizational environments and cultural backgrounds influence the outcomes of digital leadership, enhancing the model’s generalizability.

In addition, although employee creativity was rated by supervisors to reduce same-source bias, such evaluations are inherently subjective and may be influenced by personal relationships or rater bias. While efforts were made to minimize these biases, they still present a limitation in the measurement of creativity. To improve measurement validity, future studies could incorporate objective indicators of creativity (e.g., innovation outcomes, awards, or project evaluations). These indicators would provide more concrete measurements of creativity, reducing reliance on subjective ratings. Moreover, incorporating peer-based assessments could offer additional perspectives and further mitigate potential rater biases.

Finally, this study controlled for key demographic variables—age, gender, education level, work experience, and tenure—to minimize confounding influences. However, future research could extend the model by examining how organizational-level factors, such as digital maturity, innovation climate, or leadership culture, shape the relationship between digital leadership and employee creativity.

## 6. Conclusions

This study was based on social cognitive (Bandura, 2001) and social exchange (Blau, 1964) theories to develop a conceptual framework that explains the underlying mechanisms and boundary conditions of how digital leadership influences employee creativity in the digital era. The findings reveal that digital leadership enhances creativity by fostering employees’ innovation self-efficacy and encouraging knowledge sharing. Notably, building a collaborative digital environment for knowledge sharing plays a more substantial role in strengthening the effect of digital leadership on employee creativity compared to innovation self-efficacy. Moreover, employees with higher levels of technology readiness benefit more from digital leadership both in terms of strengthened psychological confidence and collaborative behavior. These findings contribute to leadership theory by demonstrating how digital leadership impacts individual psychological (SCT) and behavioral (SET) factors to shape creative outcomes in technologically dynamic workplaces. These findings offer theoretical and practical insights, guiding scholars and managers to understand how individual cognitive and social factors interact with digital leadership to shape creative outcomes in technologically dynamic workplaces.

## Figures and Tables

**Table 1:** Correlation analysis.

	Mean	SD	1	2	3	4	5	6	7	8	9	10
Digital leadership	2.95	1.11	1									
Innovation self-efficacy	3.10	1.15	0.326 **	1								
Knowledge sharing	2.95	1.07	0.399 **	0.294 **	1							
Employee creativity	3.07	1.10	0.322 **	0.27 **	0.311 **	1						
Technology readiness	3.45	1.04	0.208 **	0.237 **	0.275 **	0.346 **	1					
Gender	1.26	0.44	0.035	0.021	0.058	0.064	0.038	1				
Age	30.56	4.51	−0.128	−0.037	−0.084	0.035	−0.06	0.061	1			
Education	4.03	0.50	0.064	0.062	0.007	0.017	−0.077	−0.028	−0.132	1		
Years of work	6.74	4.24	−0.090	−0.036	−0.037	0.032	0.004	0.055	0.711	−0.220	1	
Length of service	8.00	4.60	−0.079	0.002	−0.072	0.036	−0.035	0.020	0.790	−0.200	0.694	1

Note: N = 234. ** *p* < 0.01; Gender (1 = male, 2 = female); Education level (1 = below high school, 2 = high school, 3 = college, 4 = undergraduate, 5 = master, 6 = doctorate).

**Table 2:** Confirmatory Factor Analysis (CFA) results.

	χ^2^	df	χ^2^/df	RMSEA	CFI	TLI
Five-factor model	762.160	550	1.386	0.041	0.956	0.952
Four-factor model ^a^	1208.879	554	2.182	0.071	0.864	0.854
Three-factor model ^b^	1368.089	557	2.456	0.079	0.832	0.821
Two-factor model ^c^	1589.034	559	2.843	0.089	0.787	0.773
One-factor model ^d^	2444.428	560	4.365	0.120	0.610	0.585

Note: ^a^: Digital leadership and knowledge sharing are combined into one latent factor; ^b^: Digital leadership, knowledge sharing, and employee innovation self-efficacy are combined into one latent factor; ^c^: Digital leadership, knowledge sharing, employee innovation self-efficacy, and employee creativity are combined into one latent factor; ^d^: Digital leadership, knowledge sharing, employee innovation self-efficacy, employee creativity, and employee technology readiness are combined into one latent factor.

**Table 3:** Regression analysis results.

	Innovation Self-Efficacy		Knowledge Sharing		Employee Creativity			
					Model 1 (ISE)		Model 2 (KS)	
	β	SE	β	SE	β	SE	β	SE
Intercept term	2.752	0.856	1.853	0.840	3.132	0.771	1.627	0.8455
***Control variables***								
Gender	0.040	0.160	0.110	0.144	0.114	0.154	0.096	0.154
Age	−0.009	0.028	−0.007	0.025	0.017	0.027	0.017	0.027
Education	0.108	0.133	−0.029	0.120	0.001	0.128	0.028	0.127
Work experience	−0.012	0.025	0.011	0.023	0.005	0.024	0.001	0.024
Work tenure	0.022	0.026	−0.014	0.024	−0.003	0.026	0.004	0.025
***Independent variable***								
Digital leadership	0.267 **	0.076	0.328 **	0.069	0.296 **	0.072	0.266 **	0.074
***Mediating variables***								
Knowledge sharing							0.224 **	0.069
Innovation self-efficacy					0.176 **	0.062		
***Interaction term***								
Digital leadership × Technology readiness	0.183 *	0.073	0.159 *	0.066				
***Moderating variable***								
Technology readiness	0.224 **	0.072	0.221 **	0.066				
R^2^	0.167		0.2219					
Adjusted R^2^	0.023		0.020					

Note: ** *p* < 0.01, * *p* < 0.05.

**Table 4:** Mediation and moderated mediation results.

Effects	β	SE	LLCI	ULCI
*Mediation*				
DL → ISE → EC	0.079	0.035	0.017	0.154
DL → KS → EC	0.110	0.041	0.033	0.194
*Moderated mediation*				
DL × TR → ISE → EC	0.032	0.019	0.002	0.075
DL × TR → KS → EC	0.036	0.020	0.002	0.080

Whereas DL = Digital leadership, ISE = Innovation self-efficacy, KS = Knowledge sharing, EC = Employee creativity, TR = Technology readiness.

## Data Availability

The original contributions presented in this study are included in the article. Further inquiries can be directed to the corresponding author.

## Appendix A. Measurement Scale

Digital leadership

Adapted from Zeike et al. (2019)

1. My leader is very interested in using digital technologies and tools.
2. My leader is an expert in digitalization.
3. My leader is able to keep up with the times in digital knowledge.
4. My leader is actively promoting the company’s digital transformation.
5. My leader can fully mobilize the enthusiasm of company members for digital transformation.
6. My leader has a comprehensive and clear understanding of the structure and processes required for the company’s digital transformation.

Innovation self-efficacy

Adapted from Tierney and Farmer (2002)

1. I am confident in my ability to use innovation to solve problems.
2. I think I am good at generating novel ideas.
3. I am good at developing other people’s ideas into my own.

Knowledge Sharing

Adapted from Lu et al. (2006)

1. In daily work, I actively impart business knowledge to colleagues.
2. I share useful work experience and insights with everyone.
3. After learning new knowledge that is useful for work, I promote it so that more people can learn it.
4. At work, I share my knowledge with more people.
5. I actively use the company’s existing information technology to share my knowledge.
6. As long as other colleagues need it, I always tell them everything I know.

Employee creativity

Adapted from Baer and Oldham (2006)

1. I have come up with many creative ideas to improve the working conditions of the organization.
2. I often come up with creative solutions to problems at work.
3. I come up with new ways to perform work tasks.
4. I am a good source of ideas.

Technology Readiness

Adapted from Baer and Oldham (2006)

1. New technologies help improve the quality of life.
2. Technology gives me more freedom of action.
3. Technology gives people more control over their daily lives.
4. Technology makes me more efficient in my personal life.
5. Others come to me for advice about new technologies.
6. In general, I am the first person in my circle of friends to get new technologies when they become available.
7. I can usually find new high-tech products and services without help from others.
8. I am able to keep up with the latest technological developments in my areas of interest.
9. When I get technical support from a high-tech product or service provider, I sometimes feel like I am being taken advantage of by someone who knows more than I do.
10. Technical support hotlines are not helpful because they do not explain the problem in a way that I can understand.
11. Sometimes I think that technology systems are not designed for ordinary people to use.
12. There are no manuals for high-tech products or services written in simple language.
13. People are too dependent on technology.
14. Too much technology distracts people to a harmful degree.
15. Technology reduces the quality of interpersonal relationships by reducing interpersonal interaction.
16. I do not have confidence in doing business with a place that can only be contacted online.
